# Christian Science—A Sociological Study1Christian Science. A Sociological Study. By Charles A. L. Reed, A. M., M. D., Cincinnati, O. McClellan & Co., publishers, Cincinnati. 1898. Price, 10 cents.

**Published:** 1899-01

**Authors:** 


### CHRISTIAN SCIENCE—A SOCIOLOGICAL STUDY.[1]

AN ADDRESS on this subject to a medical audience is unique. Nevertheless, under the foregoing title this distinguished author discoursed to the Northwestern Ohio Medical Society, at its recent meeting at Lima, O. Medical journals have of late given some attention in their editorial columns to this fad, but so far as we know this is the first instance where a medical society has given it attention to the extent of listening to a formal address on the subject.

While individually we might feel disposed to question the propriety of noticing this ridiculous piece of nonsense to this degree, yet it must be confessed that if it were determined to do so, it would be difficult to fit the occasion and the subject more happily than did our Ohio confrères. Moreover, we know of no physician more competent

1. Christian Science. A Sociological Study. By Charles A. L. Reed, A. M., M. D., Cincinnati, O. McClellan & Co., publishers, Cincinnati. 1898. Price, 10 cents.

to deal fairly, exhaustively, logically and eloquently with the theme than Dr. Reed. So much for the occasion, the audience and the man; now let us examine the material in some detail.

Dr. Reed, after a general introduction foreshadowing the nature of his discourse, speaks of certain aberrant forces that are at work all over the country, manifesting their effects in religio-medical cults under various names, but all of like origin and of similar purpose. Thus faith curers, natural bonesetters, hypnotists, mesmerists, clairvoyants, christian scientists, osteopaths—all spring from distorted religious and irregular medical beliefs, and all are designed as money-making schemes for the benefit of their apostles. Our author disclaims any intent to treat them unfairly; he even accords to them, one and all, honest convictions, and concedes them all to be deeply religious in their methods; then he proceeds to convict them by their own utterances (quoting from their "divine" authority) of illogical declarations, unscientific statements, and, by inference, of blasphemous dogmas. He goes even further and by the most logical deductions establishes beyond any question their cupidity, selfishness and deceptiveness and exposes the whole system as a mere money-getting scheme.

Dr. Reed, who is a profound classic scholar, gives us during his discourse a peep into the mythologic ages and tells us of the fads of those strange and mystic epochs which, curiously enough, were not unlike those of the present day. In this paragraph we are afforded pictures of theurgic medicine from the time of Zoroaster down to the Christian era, which latter, he asserts, was ushered in amidst medical superstition. Even the early centuries of this epoch abound with medical mysticism, and the later manifestations of the same tendency may be traced in the more modern astrology of Mesmer, and the immediate legerdemain of the apostles of Christian science at Lynn.

The only bit of underlying truth in the whole outfit of these various beliefs is, says our author, the curious influence we call "suggestion." "I make the declaration," to quote his words, "that the great central truth in all this supernaturalism is to be found, not in anything supernatural at all, but in an agency the most potent known to medical science." We think it a pretty strong statement to affirm that suggestion is the most potent agency known to our science, one at all events that we should not, in our present light, be willing to fully endorse, but we quite agree with him when in the closing sentence of this same paragraph he says:

"Take these alleged 'sciences,' 'healings' and 'pathies,' tear from them the gauze of idiotic 'metaphysics,' the flummery of a weird mysticism, the robes of a hypocritical and impious sacerdotalism, and there stands revealed the powerful agency of 'suggestion.'"

Dr. Reed then briefly discusses the attitude of the law to these fads, showing clearly that those who assume to deal out these forms of therapy must possess the equipment of doctors of medicine, otherwise they are liable to the penalty prescribed for illegal practitioners.

The closing paragraph is as follows :

In conclusion, permit me to say that the great profession of medicine, concerned as it is with truth and with humanity, can well afford to watch with good-natured interest these evanescent phases of ever-recurring phenomena. They are beautiful object lessons in the psychic lives of peoples. They furnish splendid demonstrations of the persistence of primitive conceptions and their modifying influence upon human life. The great clinic is now in session.

The theme is an interesting one, especially when dealt with in such a masterful fashion as that in which Dr Reed serves it up to us. His rhetoric is charming, his wit is keen, his metaphors are fascinating, and his logic is unanswerable. We could wish that every intelligent person would read this brochure, especially that every physician would possess it and make it a part of his duty in his daily professional work to preach the doctrines that it teaches. The propaganda that it inveighs against is among one of the most wicked, dangerous, even blasphemous, that has been presented for the consideration of a credulous people.

---

THE Maryland *Medical Journal* in its issue of December 3, 1898, makes some very appropriate remarks concerning the claim a medical journal has upon the medical profession of its vicinage. We quote our contemporary in part :

"The history of the progress of medical journalism in Maryland is one which should be of great interest to the physicians of Maryland. Of late years the history of medicine has been receiving more attention than it ever received before, and nothing is more fitting than that the local history of medicine should be familiar to all before that of other places and countries is taken up.

"Medical editors and publishers in the early days had no sinecure, and the lack of encouragement in so many cases is truly pathetic. At the present day, however, there is more cause for encouragement; but, as Dr. Simmons so well shows in his elaborate and well-written article, physicians of Maryland lack certain *esprit de corps*—a certain patriotism, as it were—in supporting its home journal ; and yet, as is so well known, this very journal has been the means of bringing many a young man to prominent notice who has since gained a wide reputation and no small amount of this world's goods.

" Every trade and every profession has its organ, and the prosperity of that trade or profession is usually mirrored in the writings of their respective organs. . . . . A journal becomes successful by the ability of its contributors more than by the power of its editor. A medical journal is just what its community makes it, and each physician is responsible for the welfare of the journal which is published near him. . . . . The medical profession is an asset of the profession, and its prosperity mirrors the prosperity of the profession. The physician who refuses to write for any journal on the plea of lack of time, too often means that he has lack of ability. The really busy man rarely complains of lack of time."

We have no complaint to make in regard to the support given by the profession of Buffalo and this vicinity to the JOURNAL—especially is this true of the middle-aged and older members. It has been most liberal and constant, all of which we appreciate. We do think, however, that the younger physicians are too apt to undervalue their local journal, and we commend the foregoing especially to their thoughtful consideration.

